# Supplementary material for: An open-source framework for large-scale, flexible evaluation of biomedical text mining systems
Source: J Biomed Discov Collab. 2008 Jan 29;3:1. doi: 10.1186/1747-5333-3-1 (PMC2276192; doi:10.1186/1747-5333-3-1)
Supplement: Additional file 1 — Evaluation platform source code. The additional file contains the software evaluation framework discussed in this paper. The most current release can be downloaded from SourceForge.net [1]. See the accompanying README file for instructions on installation and use. Also included in the distribution is a collection of UIMA wrappers for some commonly used BioNLP tools and annotated corpora. [file 1747-5333-3-1-S1.zip › bionlp_uima_repository-1.0.1/docs/api/edu/uchsc/ccp/util/nlp/parser/corpus/BioIECorpusParser.html]

BioIECorpusParser (Center for Computational Pharmacology Util/UIMA API)


|  |  |  |
| --- | --- | --- |
| **Overview** \| **Package** \| **Class** \| **Tree** \| **Deprecated** \| **Index** \| **Help** | |  |
| **PREV CLASS**   **NEXT CLASS** | **FRAMES**    **NO FRAMES**     **All Classes** |  |
| SUMMARY: NESTED \| FIELD \| CONSTR \| METHOD | DETAIL: FIELD \| CONSTR \| METHOD |  |


---


## edu.uchsc.ccp.util.nlp.parser.corpus Class BioIECorpusParser

```
java.lang.Object
  edu.uchsc.ccp.util.nlp.parser.corpus.CorpusParser
      edu.uchsc.ccp.util.nlp.parser.corpus.BioIECorpusParser
```

**All Implemented Interfaces:**: Iterator

---

``` public class BioIECorpusParser extends CorpusParser ```

Parser for the BioIE corpus.   
  
Output Classes:  

- protein
- rna
- generic gene

**Author:**
:   williamb

---

| **Field Summary** | |
| --- | --- |
| `static int` | `DOCUMENT_COLLECTION_ID` |


**Constructor Summary**

`BioIECorpusParser(String corpusDirectory)`
  
          The corpus directory must contain the source files and word-freak annotation files


| **Method Summary** | |
| --- | --- |
| `void` | `close()` |
| `static List<String[]>` | `getDocuments()` |
| `static Set<String>` | `getListOfBioIEPMIDs(String directory)`             output a list of the pmids in the BioIE collection |
| `static List<TextAnnotation>` | `getTextAnnotations()` |
| `boolean` | `hasNext()` |
| `static void` | `main(String[] args)`             args[0] = BioIE corpus directory (where wordfreak files reside) |
| `GenericDocument` | `next()` |
| `static void` | `parseBioIE_AnnotationFile(BufferedReader br, String documentID, String titleText, String abstractText)` |
| `static String[]` | `parseBioIE_SourceFile(BufferedReader br, int numBlankLines)`             returns a String Array of size 2  String[0] = title  String[1] = abstract  String[2] = documentID |
| `static void` | `processBioIECorpusDirectory(String directory)` |
| `static void` | `reset()` |

**Methods inherited from class edu.uchsc.ccp.util.nlp.parser.corpus.CorpusParser**

`createTextAnnotation, error, remove, warn`

**Methods inherited from class java.lang.Object**

`clone, equals, finalize, getClass, hashCode, notify, notifyAll, toString, wait, wait, wait`

**Field Detail**

### DOCUMENT\_COLLECTION\_ID

```
public static final int DOCUMENT_COLLECTION_ID
```

**See Also:**: Constant Field Values


**Constructor Detail**

### BioIECorpusParser

```
public BioIECorpusParser(String corpusDirectory)
```

:   The corpus directory must contain the source files and word-freak annotation files

    **Parameters:**: `corpusDirectory` -


**Method Detail**

### hasNext

```
public boolean hasNext()
```

:   **Specified by:**: `hasNext` in interface `Iterator` **Specified by:**: `hasNext` in class `CorpusParser`

---


### next

```
public GenericDocument next()
```

:   **Specified by:**: `next` in interface `Iterator` **Specified by:**: `next` in class `CorpusParser`

---


### close

```
public void close()
           throws IOException
```

:   **Specified by:**: `close` in class `CorpusParser`
:   **Throws:**: `IOException`

---


### reset

```
public static void reset()
```

---


### getTextAnnotations

```
public static List<TextAnnotation> getTextAnnotations()
```

---


### getDocuments

```
public static List<String[]> getDocuments()
```

---


### processBioIECorpusDirectory

```
public static void processBioIECorpusDirectory(String directory)
                                        throws IOException
```

:   **Throws:**: `IOException`

---


### getListOfBioIEPMIDs

```
public static Set<String> getListOfBioIEPMIDs(String directory)
                                       throws IOException
```

:   output a list of the pmids in the BioIE collection

    :   **Throws:**: `IOException`

---


### parseBioIE\_SourceFile

```
public static String[] parseBioIE_SourceFile(BufferedReader br,
                                             int numBlankLines)
                                      throws IOException
```

:   returns a String Array of size 2  
    String[0] = title  
    String[1] = abstract  
    String[2] = documentID  

    :   **Parameters:**: `br` - **Returns:**: **Throws:**: `IOException`

---


### parseBioIE\_AnnotationFile

```
public static void parseBioIE_AnnotationFile(BufferedReader br,
                                             String documentID,
                                             String titleText,
                                             String abstractText)
                                      throws IOException
```

:   **Throws:**: `IOException`

---


### main

```
public static void main(String[] args)
```

:   args[0] = BioIE corpus directory (where wordfreak files reside)

    :   **Parameters:**: `args` -


---


|  |  |  |
| --- | --- | --- |
| **Overview** \| **Package** \| **Class** \| **Tree** \| **Deprecated** \| **Index** \| **Help** | |  |
| **PREV CLASS**   **NEXT CLASS** | **FRAMES**    **NO FRAMES**     **All Classes** |  |
| SUMMARY: NESTED \| FIELD \| CONSTR \| METHOD | DETAIL: FIELD \| CONSTR \| METHOD |  |


---
